# Do statins prevent or promote cancer?

**DOI:** 10.3747/co.v15i2.235

**Published:** 2008-04

**Authors:** Mark R. Goldstein, Luca Mascitelli, Francesca Pezzetta

**Affiliations:** Medical Director, Fountain Medical Court, 9410 Fountain Medical Court, Suite A-200, Bonita Springs, Florida 34135, U.S.A., ***E-mail:***markrgoldstein@comcast.net; Comando Brigata Alpina “Julia”, Servizio Sanitario, 8 Via S. Agostino, Udine 33100, Italy, ***E-mail:***lumasci@libero.it; Cardiology Service, Ospedale di Tolmezzo, Tolmezzo 33028, Italy, ***E-mail:***francesca.pezzetta@libero.it

**Keywords:** Statins, regulatory T cells, elderly

The Editor, Current Oncology December 24, 2007

In their commentary, Drs. Takahashi and Nishibori^1^ discuss putative antitumour effects of statins. However, prospective data suggest that statins actually increase cancer in certain segments of the population. Additionally, new findings regarding the immunomodulatory effects of statins may explain the mechanism by which that increase occurs^2^.

Statins increase the number of regulatory T cells (Tregs) *in vivo* by inducing the transcription factor forkhead box P3^2^. Although that increase may be beneficial in stabilizing atherosclerotic plaque by reducing the effector T-cell response within the atheroma^3^, it might impair both the innate^4^ and adaptive^5^ host antitumour immune responses. Not surprisingly, the number of Tregs present in many solid tumours correlate inversely with patient survival^6^.

Indeed, analysis of large randomized statin trials demonstrate a highly significant (*p* = 0.009) inverse association between achieved low-density lipoprotein cholesterol levels and cancer^7^. Close inspection of statin trials reveal the specific populations at risk for the development of incident cancer with statin treatment. These include the elderly^8^–^10^ and people with a history of breast or prostate cancer^11^,^12^. Furthermore, statin-treated individuals undergoing immunotherapy for cancer may be at increased risk for worsening cancer^13^.

The elderly are relatively immunosuppressed and are more likely to harbour occult cancers^14^. In the prosper (Prospective Study of Pravastatin in the Elderly at Risk) trial^8^, a 3.2-year prospective study of pravastatin for cardiovascular disease prevention in the elderly (mean age at trial entry: 75 years) at high risk for cardiovascular disease, cancer incidence was significantly increased in subjects randomized to pravastatin. In fact, the increase in cancer mortality equalled in magnitude the decrease in cardiovascular disease mortality in the statin-treated patients, leaving all-cause mortality unchanged. Likewise, *post hoc* analysis of the lipid study^9^, a 6-year prospective trial of pravastatin in individuals with cardiovascular disease, revealed a significant increase in cancer incidence in the elderly subjects (age: 65–75 years) randomized to pravastatin. In a secondary analysis of the tnt (Treating to New Targets) study^10^, elderly subjects randomized to high-dose atorvastatin (80 mg daily) versus low-dose atorvastatin (10 mg daily) demonstrated a trend toward increased death, largely from an increase in cancer mortality. Therefore, the increase in incident cancer in the elderly might be dose-related. It is highly plausible that the elderly are particularly sensitive to a statin-induced increase in Tregs, further impairing their immune response to cancer.

An alarming increase in breast cancer incidence, some of which were recurrences, was seen in women randomized to pravastatin in the care trial^11^ Thereafter, cancer was an exclusion criterion in randomized statin trials. In clinical practice, however, it is not infrequent to find an association between recurrence of breast cancer and concurrent statin therapy^15^. Long-term follow-up (10 years after trial completion) of woscops (West of Scotland Coronary Prevention Study), a 5-year prospective trial of pravastatin in hypercholesterolemic men, revealed an increase in prostate cancer in the men who were randomized to pravastatin therapy^12^. That finding indicates that cancers may become evident a decade or more after treatment with statins. Treg increases have been associated with both breast and prostate cancers^16^,^17^, and therefore, it is highly plausible that the increase in cancers seen with statin therapy is related to a statin-induced increase in Tregs.

Statin therapy has been associated with tumour progression leading to radical cystectomy in patients treated for bladder cancer with bacille Calmette–Guérin immunotherapy^13^. That association may be likewise due to a statin-induced increase in Tregs, resulting in impaired host antitumour immunity.

Statin trials have typically randomized subjects free of prevalent cancers and have been about 5 years in duration. Long-term follow-up data are limited, particularly for the development of cancer. Statins are now promoted for widespread use in adults of all ages and at high doses^18^, potentially for decades. Importantly, they are used in individuals with other significant comorbidities such as cancer. Unfortunately, the post-market surveillance of drugs has been poor^19^. Because cancer is highly prevalent in the population, particularly in the elderly, a statin-induced increase in cancer incidence will likely go unrecognized.

Long-term prospective data are needed on the feasibility of statin therapy in the very elderly, the immuno-suppressed, and those with prevalent cancer. Furthermore, long-term outcome data are needed in young individuals treated with statins for prolonged time periods. Perhaps a constant increase in Tregs over years, even in the young, will weaken host antitumour immune surveillance and increase the risk for various cancers.

In conclusion, we feel that there is ample evidence that statins may promote cancer in certain segments of the population. Currently, the indications for statin therapy are based on lipoprotein levels, prevalent cardiovascular disease, other vascular risk factors, and family history^20^. Maybe it is time for a new paradigm that also includes age extremes, prevalent cancer, a past history of cancer, and overall immunocompetence.
